# Supplementary material for: Impact of Pathway Shape and Length on the Validity of the 6-Minute Walking Test: A Systematic Review and Meta-Analysis
Source: Sensors (Basel). 2024 Dec 24;25(1):17. doi: 10.3390/s25010017 (PMC11722874; doi:10.3390/s25010017)
Supplement: Supplementary file 1 [file sensors-25-00017-s001.zip › sensors-3264360-supplementary(1).pdf]

<u>Supplementary S1</u>: Literature search terms

	Vocabulaire libre	Descripteurs CINHAL	MeSH	Emtree
**Concept 1**	Walking test<br>6 minute walk test<br>6 minutes walk test<br>6 minute walking test<br>6 minutes walking test<br>Six minute walk test<br>Six minutes walking test<br>6MWT<br>6 min walk test<br>Six min walk test<br>6 min walking test<br>Six in walking test	Walking speed<br>Walking	Walk test	Six minute walk test<br>Walk test
**Concept 2**	Arrangement<br>Configuration<br>Course<br>Distance<br>Length<br>Pattern<br>Oath<br>Shape<br>Route<br>Treadmill	Treadmills	Treadmill test<br>Treadmill tests	Treadmill

Supplementary S1: (Continued)

**Concept 3**	Dimensional measurement accuracy<br>Minimal clinically important difference<br>Reproducibility of results<br>Validity of results<br>Fidelity<br>Inter rater reliability<br>Intra rater reliability<br>Minimally detectable difference<br>Reliability<br>Sensibility<br>Sensitivity<br>Specificity<br>Reproducibility<br>Responsiveness<br>Test-retest reliability<br>Validation	Discriminant Validity<br>Internal Validity<br>External Validity<br>Concurrent Validity<br>Validity<br>Construct Validity<br>Measurement Issues and Assessments<br>Outcome Assessment<br>Outcomes Research<br>Predictive Value of Tests<br>Qualitative validity<br>Reliability and Validity<br>Reproducibility of results<br>Research measurement<br>ROC curve<br>Sensitivity and specificity<br>Stability<br>Statistical analysis<br>variance analysis"	Dimensional measurement accuracy<br>Minimal clinically important difference<br>Reproducibility of results<br>Reproducibility of finding<br>Reliability and validity<br>Reliability of results<br>Validity of results	Interrater reliability<br>Intrarater reliability<br>Outcome assessment<br>Outcome research<br>Psychometry<br>Reliability<br>Validity<br>Sensibility<br>Sensitivity and specificity<br>Reproducibility<br>Test retest reliability<br>Validation study<br>Minimal clinically important difference<br>Dimensional measurement accuracy
